# Self-Care Practices for Common Colds by Primary Care Patients: Study Protocol of a European Multicenter Survey—The COCO Study

**DOI:** 10.1155/2015/272189

**Published:** 2015-09-02

**Authors:** Birgitta M. Weltermann, Biljana Gerasimovska-Kitanovska, Anika Thielmann, Juliette Chambe, Heidrun Lingner, Enzo Pirrotta, Krzysztof Buczkowski, Selda Tekiner, Slawomir Czachowski, Tamer Edirne, Andrzej Zielinski, Hülya Yikilkan, Tuomas Koskela, Ferdinando Petrazzuoli, Robert D. Hoffman, Marija Petek Šter, Clara Guede Fernández, Ayşegül Uludağ, Kathryn Hoffmann, Vildan Mevsim, Sanda Kreitmayer Pestic

**Affiliations:** ^1^Institute for General Medicine, Essen University Hospital, University of Duisburg-Essen, Hufelandstraße 55, 45147 Essen, Germany; ^2^Department of Family Medicine and Department of Nephrology, Ss. Cyril and Methodius University of Skopje, Vodnjanska Street 17, Skopje, Macedonia; ^3^Department of General Practice, University of Strasbourg, 2a rue de Brantôme, 67100 Strasbourg, France; ^4^Centre for Public Health and Healthcare, Hannover Medical School, Carl-Neuberg-Straße 1, 30625 Hannover, Germany; ^5^SNAMID, Italian Society of General Practitioners and ASL Roma B, Via Tuscolana, 859 Rome, Italy; ^6^Department of Family Medicine, Nicolaus Copernicus University, Skłodowskiej-Curie 9, 85-094 Bydgoszcz, Poland; ^7^Department of Family Medicine, Ibni Sina Hospital, Ankara University School of Medicine, Samanpazarı, 06100 Ankara, Turkey; ^8^Department of Family Medicine, University of Pamukkale, PAU Tip Fakultesi, Aile Hekimligi AD, Kinikli Kampus, Denizli, Turkey; ^9^Blekinge Centre of Competence and Lyckeby Primary Health Care Centre, Källevägen 12, 37162 Lyckeby, Sweden; ^10^Family Medicine Department, Dışkapı Yıldırım Beyazıt Training and Research Hospital, Irfan Basbug Caddesi, Dışkapı, 06110 Ankara, Turkey; ^11^Department of General Practice, University of Tampere, Lääkärikatu 1, 33014 Tampere, Finland; ^12^Department of Clinical Sciences in Malmö, Centre for Primary Health Care Research, Lund University, Malmö, Sweden; ^13^Departments of Family Medicine and Medical Education, Saklar Medical School, Tel Aviv University, 8 Gordon Street, 76291 Rehovot, Israel; ^14^Department of Family Medicine, University of Ljubljana, Poljanski Nasip 58, SI-1000 Ljubljana, Slovenia; ^15^EOXI Vigo, University of Vigo, Calle Zaragoza, Vigo, 36203 Pontevedra, Spain; ^16^Department of Family Medicine, Faculty of Medicine, Çanakkale Onsekiz Mart University, Terzioğlu Campus, 17100 Çanakkale, Turkey; ^17^Department of General Practice and Family Medicine, Center for Public Health, Medical University of Vienna, Kinderspitalgasse 15, 1st Floor, 1090 Vienna, Austria; ^18^Department of Family Medicine, Dokuz Eylül University Faculty of Medicine, Izmir, Turkey; ^19^Medical Faculty Tuzla, Family Medicine Department and Health Center Tuzla, Department for General/Family Medicine, Marsala Tita 199, 75000 Tuzla, Bosnia and Herzegovina

## Abstract

*Background.* Self-care for common colds is frequent, yet little is known about the spectrum, regional differences, and potential risks of self-care practices in patients from various European regions. *Methods/Design.* We describe the study protocol for a cross-sectional survey in 27 primary care centers from 14 European countries. At all sites, 120 consecutive adult patients, who visit their general practitioner for any reason, filled in a self-administered 27-item questionnaire. This addresses patients' self-care practices for common colds. Separately, the subjective level of discomfort when having a common cold, knowing about the diseases' self-limited nature, and medical and sociodemographic data are requested. Additionally, physicians are surveyed on their use of and recommendations for self-care practices. We are interested in investigating which self-care practices for common colds are used, whether the number of self-care practices used is influenced by knowledge about the self-limited nature of the disease, and the subjective level of discomfort when having a cold and to identify potential adverse interactions with chronic physician-prescribed medications. Further factors that will be considered are, for example, demographic characteristics, chronic conditions, and sources of information for self-care practices. All descriptive and analytical statistics will be performed on the pooled dataset and stratified by country and site. *Discussion.* To our knowledge, COCO is the first European survey on the use of self-care practices for common colds. The study will provide new insight into patients' and general practitioners' self-care measures for common colds across Europe.

## 1. Introduction

Worldwide, common colds are the most frequently encountered human disease [1]. Common cold is a conventional term for a heterogeneous group of mild upper respiratory illnesses caused by more than 100 viruses such as rhinoviruses, RSVs, influenza A viruses, adenoviruses, and parainfluenza type 3 viruses [2, 3]. The incidence of common colds is known to be age-specific [4], with a yearly average of 6–8 episodes in younger children decreasing to 2–4 episodes in adulthood [5, 6]. According to a US American study (2015) with 3333 participants, 85% of the population above 18 years of age will develop at least 1 common cold per year, lasting between 3 and 7 days [7]. To our knowledge, no European data are available. The incidence for the European region is likely comparable. The socioeconomic costs caused by common colds are a burden for society, especially costs due to sick leaves [8]. The evidence for common cold treatments is poor with only few medications proven effective in relieving symptoms or in reducing the duration of the disease, for example, nonsteroidal anti-inflammatory drug [9], oral antihistamine-decongestant-analgesic combinations [10], nasal decongestants [11], and zinc (lozenges or syrup) [8]. Despite poor evidence, systematic and unsystematic observations show that patients use a wide variety of self-care practices for this self-limited disease [12–16].

Following the definition of the WHO [17], this study understands self-care as “(…) the ability of individuals, families and communities to promote health, prevent disease, and maintain health and to cope with illness and disability (…). It is a broad concept encompassing (…) nutrition (…), lifestyle (…) (and) self-medication.” Thus, self-care practices involve all patient driven health actions, including the dimensions: self-medication, complementary medicine, and the so-called home remedies [16].

Until now, no study exists that compares self-care practices for common colds in different European countries. The topic is of interest for several reasons: (1) the spectrum of self-care practices used for common colds is unknown; (2) potential medication interactions in patients on chronic, physician-prescribed medications have not been evaluated; (3) factors which influence the use of self-care practices are poorly understood. Yet, it may be important to better understand these topics because the same factors may drive the high demand and overprescription of antibiotics for common colds, despite public campaigns informing about the benign and self-limited disease course. The European working group on self-care was formed to address these issues. The group consists of members of the European General Practice Research Network (www.egprn.org).

## 2. Methods and Materials

The primary aim of this study is to determine which self-care practices for common colds are used by primary care patients from different European countries and to identify factors influencing self-care practices. To investigate self-care practices for common colds, we are conducting a multicenter cross-sectional survey in 14 European countries.

### 2.1. Hypotheses

The study will investigate the following specific hypotheses:

1. There are differences in the use of self-care practices for common colds between countries (Purchasing Power Standard, region) and within countries (rural/urban areas).
2. There are differences in the use of self-care practices according to patients' socioeconomic factors (age, gender, migration background, health insurance status, and number of school years (including higher education)), knowing about the self-limited nature of common colds, subjective level of discomfort when having a common cold, lifestyle factors (smoking), chronic conditions, number of tablets taken daily, and sources of information.
3. The use of self-care practices in the subgroup of patients on chronic physician-prescribed medications bears the risk of potential adverse effects due to interactions between self-care practices and medications. This risk evaluation will be performed using a pharmacological review on interactions between, for example, warfarin, coumadin, ASA/aspirin, and oral contraceptives/“the pill” (e.g., warfarin and licorice, ibuprofen and willow, contraceptives, and antidepressants such as amitriptyline and SSRI with St. John's wort) [18].

In addition to these specific hypotheses, the study will explore self-care practices of participating general practitioners: which self-care practices do they apply themselves and which do they recommend to their patients?

### 2.2. Coordination and Participating Sites of the COCO Study

This cross-sectional study was initiated by primary care physician researchers from three European countries (Germany, Macedonia, and Bosnia and Herzegovina) during a conference of the European General Practice Research Network (EGPRN) in October 2012. The steering committee used subsequent EGPRN meetings in 2013 to recruit additional academic primary care physicians from the EGPRN as working group partners. The final working group consists of 25 EGPRN members from 14 European and associated countries: Austria, Bosnia and Herzegovina, Finland, France, Germany, Israel, Italy, Macedonia, Poland, Romania, Slovenia, Spain, Sweden, and Turkey. At each site, the coordinators are responsible for the translation of the English questionnaire into the site language, its distribution to patients, the back-translation of patient's answers to open questions into English, and either the transfer of the questionnaire for data entry at the study center in Essen, Germany, or the data entry on-site with subsequent transmission of the dataset to the study center.

The coordinators at each site signed a consent form for the outline of the study plan and details on expected participation requirements, that is, the translation of the questionnaire, the sampling process, and the data handling. Coordinators are responsible for following the study plan.

The first ethical approval was obtained from the Ethics Committee of the University of Duisburg-Essen, Germany (13-5495-BO). This ethical approval was provided to the coordinators of all participating sites who obtained any approvals required, including, if necessary, an additional ethical approval according to local laws and guidelines.

### 2.3. Study Instruments and Questionnaire Design Process

The questionnaire was developed based on a preceding survey in 10 primary care physicians from seven European countries and one associated country (Austria, Bosnia and Herzegovina, Germany, Israel, Italy, Macedonia, Poland, and Turkey). Physicians were asked to name typical self-care items used by their patients for common cold [19]. The physicians' answers were grouped to construct the survey instrument. The final questionnaire consists of 27 questions with a total of 94 self-care items in 11 categories: over-the-counter medication (11 items), specific food or drinks (11 items), herbal tea (18 items), alcoholic drink (3 items), self-prepared special recipe (7 items), pastilles or drops (10 items), something for the nose (4 items), inhalation (8 items), gargle or spray for the throat (4 items), something external (5 items), and extras at home (13 items). For each category, an additional free-text option is provided. The questionnaire approach of a combination of closed and open questions was chosen because participants are more likely to recall treatments when closed or semiclosed product-specific questions are provided rather than a single open question only [20].

Furthermore, the questionnaire elicits the following patient characteristics: age, gender, place of birth, origin of family, health insurance status, number of school years, number of pills taken daily, regular intake of specific medications (i.e., anticoagulants, birth control, and aspirin) daily smoking, money spent for the last common cold, source of information for self-care practices, whether self-care practices were recommended to others, and having one of the following chronic conditions: depression, chronic kidney disease, chronic pain/arthritis, asthma/chronic bronchitis, high blood pressure, heart disease, and diabetes. Following the Common Sense Self-Regulation Model (CS-SRM) [21], the subjective level of discomfort is measured asking whether one “feels very bad when having a common cold” (answer options: “yes,” “no,” and “do not know”). The perception regarding control and cure is measured focusing on the self-limitedness of the disease “if a common cold goes away by itself” (answer options: “yes,” “no,” and “do not know”). Rather than providing a detailed definition for common colds, we relied on the participants' intuitive understanding as laymen of what is meant by common colds. The complete patient questionnaire is displayed in additional file 1 in Supplementary Material available online at http://dx.doi.org/10.1155/2015/272189.

After a pretest in 10 primary care patients each in Macedonia and Germany as well as a working group discussion at the fall EGRPN meeting in Kuşadası, Turkey, the questionnaire was finalized. The original questionnaire was developed in English. Each study site coordinator is responsible for its translation into the native languages and its review by a second physician.

For data analysis, the categories are reclassified according to the mode of application to avoid overlap between groups: oral application, intranasal application, inhalation, topical use in throat, external use on the body, and extras at home. Foodstuffs are treated as a separate category, although formally they would belong to the oral application group. All industry-prepared items from the pharmacy/drug store which require no prescription are grouped as over-the-counter (OTC) medications.

At all sites, a short questionnaire collects characteristics of the participating practices and physicians: number of physicians in practice, total number of patients in the previous year, average patient contacts per day by the physician, age distribution of patients in the previous year, percentage of patients with migration background, and whether the practice is located in an urban or rural area. For all participating physicians, the number of years working in the practice, the three typical self-applied measures for colds, and the three most frequently recommended measures for patients are obtained (see additional file 2).

### 2.4. Data Collection and Management

#### 2.4.1. Sampling Procedure

Each site coordinator was instructed how to perform the standardized questionnaire translation and the data collection in the selected primary care practice(s) or center(s). To ensure a random sample, questionnaires are distributed to consecutive patients fulfilling the inclusion criteria, independent of their reason for the practice visit. The inclusion criteria are age above 18 years and sufficient language capabilities to answer the self-administered questionnaire or being in attendance of someone able to provide assistance (in reading, translating, or other support). The site coordinator organizes the distribution of the questionnaires at the reception desk to be completed by the patient in the waiting room. In group practices or centers with several physicians, questionnaires will be distributed to consecutive patients, regardless of the physician in charge of the patient.

To calculate practice response rates, refused or unfilled questionnaires are collected together with the completed questionnaires. Details about the sampling process in each participating site (e.g., response rate per site) will be included in the main publication.

#### 2.4.2. Data Management

Data entry is organized using IBM SPSS Statistics for Windows, Version 22.0. Participating sites are free to choose between uploading the data into a custom-designed SPSS data file or having the data entry arranged at the study center in Essen, Germany. In the latter case, participating sites either uploaded scanned anonymous questionnaires to a secured university server drop box or mailed the original questionnaires using a certified mail service. To ensure data quality, 10% of the data entered in Essen are checked through double entry by a second person. If errors are greater than 5%, the complete questionnaire is double entered.

#### 2.4.3. Data Quality

Plausibility checks for contradictory answers are performed. The number of pills taken daily is adjusted if a person marks “0” but answers that they take, for example, a contraceptive. Free-text answers are checked if: (a) an answer is only given as free-text though provided in a multiple choice format (correction: free-text answers deleted and multiple choice question marked); (b) the same answer is given as in the multiple choice question (correction: free-text item deleted); (c) answers are given in the wrong category (correction: rearranged). All currencies are transferred into Euro.

### 2.5. Sample Size Calculation and Statistical Analysis

#### 2.5.1. Sample Size Calculation

The targeted sample size for each site was estimated from power calculations using German data on nonsteroidal analgesic use rates because no data were available on the utilization rate of self-care practices for all participating countries. With 62 million packages used by 80 million people, about 77% of the population used such medications in 2012 (IMS PharmaScope National by IMS HEALTH GmbH & Co. OHG). Assuming that some patients use more than one package per year, we estimated that about 40% of the adult population took nonsteroidal painkillers such as acetylsalicylic acid, ibuprofen, or paracetamol at least once a year.

Aiming at estimating the sample size necessary to obtain a representative sample for a practice with 3,000 patients, a total of 94 patients need to be studied (CI 95%; SE 0.05). In a practice sample of 1,000 patients with all other parameters kept equal, a total of 88 patients would need to be surveyed. Similarly, in a practice with 10,000 patients, a total of 96 patients would need to complete the questionnaire. These estimates are based on two assumptions. (1) The sample of patients surveyed is random for the respective practice. (2) The sample size of 100 patients is a tradeoff between statistical power and practicability. To account for a nonresponse rate of 25%, oversampling by 25% is planned, leading to an adjusted sample size of 117.5 (94 + 23.5 patients), rounded to 120 patients per primary care practice or center.

#### 2.5.2. Response plus Representativeness

The response is calculated as percentage of the questionnaires filled out in relation to the 120 questionnaires distributed. Due to data protection reasons, no characterization of nonresponders is possible. Analyses of full and partial responders will be performed.

The power calculation focuses on the representativeness of the samples on practice level. In addition, we will investigate whether the collected data are also representative on a country level. To address this issue, we are using four analytic approaches:

(1) In countries with ≥2 samples from different sites within one city and its adjacent suburbs, frequencies of self-care items will be compared between the different sites (France, Turkey). Thereby, different socioeconomic and geographical areas (rural/urban) will be compared.
(2) In countries with ≥2 different cities, the frequencies of self-care items will be compared between the different cities (Germany, Turkey, and Poland). In one case, we will compare a practice sample (*n* = 120) with a nationally representative sample obtained through telephone interviews conducted on the basis of the COCO questionnaire (Austria).
(3) Due to demographic heterogeneity between countries, we will perform age and if needed sex standardization.
(4) Standardization for regional characteristics (urban/rural) will be considered as well.

#### 2.5.3. Pooled and Country Specific Analyses

In preparation for pooled analyses across 14 countries, potential sources of heterogeneity within the data will be identified before combining the datasets.

All descriptive statistics will be performed on the pooled dataset and stratified by country and site. The prevalence of items used will be presented as proportions.

We will use chi-square tests for categorical and ANOVA for continuous variables to identify factors influencing self-care items (age, sex, urban/rural area, number of school years, migration background, health insurance status, subjective level of discomfort, knowing about the diseases' self-limited nature of common colds, self-growing of plants/herbs, smoking, chronic conditions, number of tablets taken daily, and different sources of information). Analyses according to the Purchasing Power Standard [22] will be performed on the basis of the following groupings: Group I: Austria, Sweden, Germany, and Finland; Group II: France, Israel, Italy, Spain, and Slovenia; Group III: Poland, Turkey, Romania, Macedonia, and Bosnia and Herzegovina. Differences between geographical region, rural/urban area, and cultural background will be tested using the chi-square statistic. For the comparison of more than two means (Purchasing Power Standard), ANOVA will be calculated. Logistic regression analyses will be used to determine predictors for self-care practices. All analyses will be performed in the total dataset and separately per site and country and European region.

## 3. Discussion

The COCO study aims at describing self-care practices for common colds used by primary care patients in different European countries to identify the spectrum used, to quantify self-care practices, to describe differences across countries and regions, and to explore the risk of possible interactions with chronic physician-prescribed medications. Interestingly, despite their high prevalence and their impact on individuals and societies, common colds and especially self-care practices for this harmless disease have barely been a subject of interest for the medical community.

In contrast to recent prior studies on self-care, which mostly focus on single dimensions, that is, self-medication or home remedies [12, 23], this study explores self-care for common colds based on the comprehensive WHO definition, including OTC and nonmedicinal home remedies. This broad WHO definition is chosen because of the poor prior knowledge on the spectrum of items used in the participating countries, cultural differences, and the expected differences due to healthcare systems. In order to survey the likely range of self-care practices used in the participating countries and because of the lack of a standardized questionnaire, the questionnaire design chosen includes closed questions and allows for free-text answers.

This pan-European multicenter study is conducted using a low budget strategy, thereby representing an example for future projects. Nevertheless, there are several methodological challenges and limitations to this study: First, although the high number of participating sites throughout Europe is a strength of the COCO study, this project is facing a major methodological challenge with regard to the representativeness of its results on practice, local, regional, and national level. This challenge will be addressed by combining various analytic strategies: Depending on the number of individual samples within a country, frequencies of self-care items will be compared either between different areas of the same city or between different cities within one country. An additional nationally representative sample obtained through telephone interviews on the basis of the COCO questionnaire allows for a comparison of single primary care practices and a national sample. Further, age and sex standardization as well as standardization for regional characteristics will be considered. Second, this cross-sectional questionnaire survey is based on the patients' recall of self-care practices during their last common cold. This may imply a declaration and recall bias. In order to limit those, the questionnaire is distributed at the end of winter time during the typical peak season for common colds. Also, it is unlikely that patients completely change self-care practices from one common cold to another, thus implying rather stable answering behavior. Furthermore, the questionnaire was only two pages long and included product-specific questions. This questionnaire design has been proven to overcome the recall bias for treatments [20].

## 4. Conclusion

The COCO study will provide insight into patients' self-care behaviors for common colds. The results not only will be interesting from a descriptive perspective with regard to regional differences within Europe, but will also help to guide research on educational interventions on the harmlessness of common colds and appropriate self-care practices. Also, the results will help to develop strategies to better protect patients on chronic medications from adverse medication interactions due to self-care practices for common colds.

## Supplementary Material

The supplementary material contains two files: file 1 shows the self-administered patient questionnaire addressing self-care for common cold; file 2 shows the physician questionnaire which was filled in by all collecting sites.

## Abbreviations

COCO: Common Cold study
WHO: World Health Organization.

## Trial Status

Participants' recruitment finished in April 2014.
