# Supplementary material for: Self-Care Practices for Common Colds by Primary Care Patients: Study Protocol of a European Multicenter Survey—The COCO Study
Source: Evid Based Complement Alternat Med. 2015 Sep 2;2015:272189. doi: 10.1155/2015/272189 (PMC4572439; doi:10.1155/2015/272189)
Supplement: Supplementary file 1 — The supplementary material contains two files: file 1 shows the self-administered patient questionnaire addressing self-care for common cold; file 2 shows the physician questionnaire which was filled in by all collecting sites. [file 272189.f1.pdf]

Additional file 1

Study information & Questionnaire

European study about patients’ self-care for common colds
“What do you do when having a common cold?”

Common colds are frequent. Various self-medications and home remedies are used to relieve symptoms. Our practice is participating in a study of the European Network of General Practitioners: physicians from more than 15 European universities are studying what patients use for common colds.

We like to ask for your participation: please complete this questionnaire. The data collection is anonymous, so that you as a person cannot be identified. The results will be published scientifically.

Please mark: When you had a common cold during the last year: what did you do?

1. I took over-the-counter medication: No □$_0$ Yes □$_1$, please specify:

□$_1$ ASA/aspirin	□$_1$ Paracetamol	□$_1$ Ibuprofen
□$_1$ Vitamin C	□$_1$ Calcium	□$_1$ Zinc
□$_1$ Cough syrup	□$_1$ Cough drops	□$_1$ “Painkillers“
□$_1$ Homeopathic drugs	□$_1$ “Pills for colds”	□$_1$ Other: _______________

2. I ate or drank the following food: No □$_0$ Yes □$_1$, please specify:

□$_1$ Orange (juice)	□$_1$ Lemon (juice)	□$_1$ Grapefruit (juice)	□$_1$ Chicken soup
□$_1$ Plenty of water	□$_1$ Honey	□$_1$ Garlic	□$_1$ Food with marjoram
□$_1$ Mandarins	□$_1$ More fruits	□$_1$ More vegetables	□$_1$ Other: ____________

3. I prepared a herbal tea: No □$_0$ Yes □$_1$, please specify:

□$_1$ Thyme	□$_1$ Camphor	□$_1$ Eucalyptus	□$_1$ Elderberry
□$_1$ Ginger	□$_1$ Pine syrup	□$_1$ Chamomile	□$_1$ Echinecea propolis
□$_1$ Cinnamon	□$_1$ Peppermint	□$_1$ Cranberry	□$_1$ Mixed herbal tea
□$_1$ Sage	□$_1$ St. Johns Wort	□$_1$ Black tea	□$_1$ Other: ____________
□$_1$ Raspberry	□$_1$ Marshmallow root	□$_1$ Lime blossom	

4. I used an alcoholic drink: No □$_0$ Yes □$_1$, please specify:

□$_1$ Mulled wine	□$_1$ Tea with rum
□$_1$ Amber with alcohol	□$_1$ Other: _______________

5. I prepared a special recipe: No □$_0$ Yes □$_1$, please specify:

□$_1$ Tea from apple peel	□$_1$ Onion (juice) with honey	□$_1$ Hot water with lemon & honey
□$_1$ Hot milk with butter	□$_1$ Hot milk with honey	□$_1$ Other: ____________
□$_1$ Onion cough syrup	□$_1$ Black radish with sugar	

6. I used pastilles or drops: No □$_0$ Yes □$_1$, please specify:

□$_1$ Lemon	□$_1$ Cinnamon	□$_1$ Peppermint	□$_1$ Plantago cough syrup
□$_1$ Sage	□$_1$ Curcuma	□$_1$ Eucalyptus	□$_1$ Other: ____________
□$_1$ Herbal pastilles	□$_1$ Umkaloabo	□$_1$ Cough pastilles	

7. I used something for my nose: No □$_0$ Yes □$_1$, please specify:

□$_1$ Nasal decongestant	□$_1$ Saline nasal spray	□$_1$ Other: ____________
□$_1$ Nasal „washings“	□$_1$ Other nose drops/ spray	

EGPRN working group on self-care | Steering committee: PD Dr. Weltermann, University of Duisburg-Essen, Germany; Prof. Gerasimovska, University St. Cyril and Methodius, Macedonia; Dr. Hasanagic, Association of Family Physicians, Federation of Bosnia and Herzegovina.
Contact: Institute for General Medicine, University of Duisburg-Essen, Germany, Phone: +49-201-877-869-0, Fax: -20

8. I used an inhalation: No □$_0$ Yes □$_1$, please specify:

□$_1$ Humidifier □$_1$ Pot with steaming water □$_1$ Chamomile

□$_1$ Eucalyptus (oil) □$_1$ Saline □$_1$ Essential oil

□$_1$ Peppermint □$_1$ Camphor (oil) □$_1$ Other: ____________

9. I used a gargle or spray for my throat: No □$_0$ Yes □$_1$, please specify:

□$_1$ Gargle with saline □$_1$ Gargle with sage □$_1$ Other: ____________

□$_1$ Gargle with chamomile □$_1$ Throat spray

10. I used something externally on my body: No □$_0$ Yes □$_1$, please specify:

□$_1$ Vacuum cupping □$_1$ Chest rub with alcohol □$_1$ Essential oil rub

□$_1$ Fire cupping □$_1$ Mentholated cream □$_1$ Other: ____________

11. At home I did some things extra: No □$_0$ Yes □$_1$, please specify:

□$_1$ Stayed in bed □$_1$ Hot bath or shower □$_1$ Cold shower

□$_1$ Took a day/ days off □$_1$ Prayed for recovery □$_1$ Calf compresses

□$_1$ Hot water bottle □$_1$ Electric warming blanket □$_1$ Hot bath with lime blossom

□$_1$ Opened windows □$_1$ Warm covers □$_1$ Other: ____________

□$_1$ Warm clothes □$_1$ Rest at home

12. Who taught you about these measures? □$_1$ Parents □$_1$ Other family members

□$_1$ Friends, neighbors □$_1$ Physician □$_1$ Pharmacy □$_1$ Newspaper, internet

13. Did you recommend these measures to others? □$_0$ No □$_1$ Yes □$_2$ Don´t know

14. Your opinion: A common cold goes away by itself: □$_0$ No □$_1$ Yes □$_2$ Don´t know

15. Do you feel very uncomfortable when having a cold: □$_0$ No □$_1$ Yes □$_2$ Don´t know

16. Your age: _____ (in years)

17. Your gender: □$_0$ Male □$_1$ Female

18. Your health insurance is: □$_0$ Public □$_1$ Private

19. Number of pills you take daily: ______

20. Which chronic conditions do you have? *Please mark all that apply.* □$_1$ Depression

□$_1$ Chronic kidney disease □$_1$ Chronic pain / Arthritis □$_1$ Asthma/Chronic bronchitis

□$_1$ Diabetes □$_1$ Heart disease □$_1$ High blood-pressure

21. Do you take any of the following medications? □$_1$ Birth control pill

□$_1$ Anticoagulants (e.g. warfarin, coumadin) □$_1$ ASA/aspirin daily for heart attack/stroke

22. Do you use self-grown plants for common colds? No □$_0$ Yes □$_1$

23. For the last cold, you spent about: _____ Euros

24. How many years did you go to school? (including higher education) ____ Years

25. Do you smoke daily? □$_0$ No □$_1$ Yes

26. Country where you were born: ________ **27. Country your family stems from:** ________

THANK YOU VERY MUCH!

EGPRN working group on self-care | Steering committee: PD Dr. Weltermann, University of Duisburg-Essen, Germany; Prof. Gerasimovska, University St. Cyril and Methodius, Macedonia; Dr. Hasanagic, Association of Family Physicians, Federation of Bosnia and Herzegovina.
Contact: Institute for General Medicine, University of Duisburg-Essen, Germany, Phone: +49-201-877-869-0, Fax: -20

Additional file 2

EGPRN study about self-care for common colds
“What do you do when having a common cold?”

Dear colleague,

Please answer the following questions about yourself and your data collection site:

1. **Please specify the name and address of the practice collecting your data:**
   ____
   ____
   ____

2. **How long do you work in this practice?**
   □$_0$ 0-5 years □$_1$ 6-15 years □$_2$ 16-25 years □$_3$ More than 25 years
   □$_4$ I do not work in this practice

3. **Number of physicians in your practice:**
   ___ Full-time physicians
   ___ Part-time physicians

4. **How many patients do you see in this practice per day on average?**
   □$_0$ <10 □$_1$ 11-15 □$_2$ 16-25 □$_3$ 26-40 □$_4$ 41-60 □$_5$ 61-80 □$_6$ ≥81

5. **What is the total number of patients served in this practice in 2012?** ______

6. **To compare your practice with other participating sites: How many patients did this practice see in 2012 (in total numbers)?**
   < 18 years: ______patients 18-40 years: _____patients 41-65 years: _____patients
   66-80 years: _____patients ≥ 81 years: _____patients

7. **Please estimate the percentage of patients with migration history in your practice? ___ %**

8. **Is your practice located in an urban or rural area?** □$_0$ Urban □$_1$ Rural □$_2$Mixed

9. **What do you typically <u>take or do yourself</u> when having a common cold?**
   Please list up to three things.
   1. ________________________
   2. ________________________
   3. ________________________

10. **What do you typically <u>recommend or prescribe to patients</u> with a common cold?**
    Please list up to three things.
    1. ________________________
    2. ________________________
    3. ________________________

Thank you very much!

EGPRN working group on self-care | Steering committee: PD Dr. Weltermann, University of Duisburg-Essen, Germany; Prof. Gerasimovska, University St. Cyril and Methodius, Macedonia; Dr. Hasanagic, Association of Family Physicians, Federation of Bosnia and Herzegovina.
Contact: Institute for General Medicine, University of Duisburg-Essen, Germany, Phone: +49-201-877-869-0, Fax: -20
